# Supplementary material for: Modeling glioblastoma heterogeneity as a dynamic network of cell states
Source: Mol Syst Biol. 2021 Sep 16;17(9):e10105. doi: 10.15252/msb.202010105 (PMC8444284; doi:10.15252/msb.202010105)
Supplement: Supplementary file 6 — Source Data for Figure 5 [file MSB-17-e10105-s004.zip › Figure5A_sourcedata/GSEA_3071/nosigdb_stateII.GseaPreranked.1622795151171/RICHARDS_NOWAKOWSKI_SCIENCE_2017_IN_CTX_MGE1_UPREG.html]

Details for gene set RICHARDS\_NOWAKOWSKI\_SCIENCE\_2017\_IN\_CTX\_MGE1\_UPREG[GSEA]

|  || Dataset | state23071 |
| Phenotype | NoPhenotypeAvailable |
| Upregulated in class | na\_neg |
| GeneSet | RICHARDS\_NOWAKOWSKI\_SCIENCE\_2017\_IN\_CTX\_MGE1\_UPREG |
| Enrichment Score (ES) | -0.5006871 |
| Normalized Enrichment Score (NES) | -3.0237696 |
| Nominal p-value | 0.0 |
| FDR q-value | 0.0 |
| FWER p-Value | 0.0 |
Table: GSEA Results Summary

  

Fig 1: Enrichment plot: RICHARDS\_NOWAKOWSKI\_SCIENCE\_2017\_IN\_CTX\_MGE1\_UPREG      
 Profile of the Running ES Score & Positions of GeneSet Members on the Rank Ordered List

  

| PROBE | GENE SYMBOL | GENE\_TITLE | RANK IN GENE LIST | RANK METRIC SCORE | RUNNING ES | CORE ENRICHMENT || 1 | ATAD5 |  |  | 18 | 1.469 | 0.0107 | No |
| 2 | RANBP1 |  |  | 149 | 0.958 | -0.0123 | No |
| 3 | DKC1 |  |  | 268 | 0.789 | -0.0340 | No |
| 4 | PARP2 |  |  | 451 | 0.650 | -0.0734 | No |
| 5 | TENM2 |  |  | 555 | 0.599 | -0.0933 | No |
| 6 | RIMS1 |  |  | 776 | 0.506 | -0.1438 | No |
| 7 | NTN4 |  |  | 883 | 0.474 | -0.1657 | No |
| 8 | CASP2 |  |  | 884 | 0.474 | -0.1608 | No |
| 9 | RAD1 |  |  | 926 | 0.464 | -0.1664 | No |
| 10 | ACKR3 |  |  | 962 | 0.454 | -0.1705 | No |
| 11 | CHML |  |  | 1043 | 0.429 | -0.1864 | No |
| 12 | PDCD5 |  |  | 1051 | 0.428 | -0.1837 | No |
| 13 | TXN |  |  | 1087 | 0.419 | -0.1882 | No |
| 14 | ZNF714 |  |  | 1103 | 0.416 | -0.1877 | No |
| 15 | CCT2 |  |  | 1108 | 0.415 | -0.1844 | No |
| 16 | SNRPF |  |  | 1150 | 0.407 | -0.1906 | No |
| 17 | PSMC3 |  |  | 1241 | 0.389 | -0.2093 | No |
| 18 | STMN1 |  |  | 1249 | 0.387 | -0.2071 | No |
| 19 | DAP3 |  |  | 1305 | 0.377 | -0.2171 | No |
| 20 | LRRC8B |  |  | 1472 | 0.344 | -0.2556 | No |
| 21 | DNTTIP2 |  |  | 1478 | 0.343 | -0.2533 | No |
| 22 | TRERF1 |  |  | 1530 | 0.335 | -0.2628 | No |
| 23 | PXDC1 |  |  | 1566 | 0.330 | -0.2682 | No |
| 24 | TPM4 |  |  | 1578 | 0.328 | -0.2676 | No |
| 25 | RFC1 |  |  | 1579 | 0.328 | -0.2642 | No |
| 26 | BCAS2 |  |  | 1631 | 0.319 | -0.2738 | No |
| 27 | CDC123 |  |  | 1647 | 0.317 | -0.2743 | No |
| 28 | PTBP3 |  |  | 1700 | 0.311 | -0.2843 | No |
| 29 | MTF2 |  |  | 1710 | 0.309 | -0.2834 | No |
| 30 | PSMG2 |  |  | 1723 | 0.307 | -0.2832 | No |
| 31 | TARS |  |  | 1730 | 0.307 | -0.2816 | No |
| 32 | EFTUD2 |  |  | 1795 | 0.299 | -0.2947 | No |
| 33 | EP400 |  |  | 1807 | 0.298 | -0.2944 | No |
| 34 | RAB8A |  |  | 1857 | 0.291 | -0.3038 | No |
| 35 | PSMD1 |  |  | 1993 | 0.273 | -0.3351 | No |
| 36 | MYL12B |  |  | 2035 | 0.269 | -0.3427 | No |
| 37 | AZIN1 |  |  | 2068 | 0.265 | -0.3481 | No |
| 38 | PSMD11 |  |  | 2129 | 0.259 | -0.3606 | No |
| 39 | KALRN |  |  | 2182 | 0.254 | -0.3712 | No |
| 40 | PCDH19 |  |  | 2279 | -0.257 | -0.3928 | No |
| 41 | LRRTM2 |  |  | 2283 | -0.257 | -0.3909 | No |
| 42 | NBEA |  |  | 2334 | -0.263 | -0.4008 | No |
| 43 | NDN |  |  | 2389 | -0.270 | -0.4117 | No |
| 44 | COX7B |  |  | 2400 | -0.271 | -0.4115 | No |
| 45 | SNX27 |  |  | 2435 | -0.275 | -0.4172 | No |
| 46 | RUNX1T1 |  |  | 2458 | -0.278 | -0.4199 | No |
| 47 | IFT52 |  |  | 2472 | -0.280 | -0.4203 | No |
| 48 | ZNF638 |  |  | 2536 | -0.291 | -0.4332 | No |
| 49 | HIVEP3 |  |  | 2554 | -0.294 | -0.4345 | No |
| 50 | DYNLRB1 |  |  | 2559 | -0.295 | -0.4325 | No |
| 51 | COX6A1 |  |  | 2629 | -0.305 | -0.4468 | No |
| 52 | KIAA1191 |  |  | 2645 | -0.307 | -0.4474 | No |
| 53 | ANKRD50 |  |  | 2653 | -0.309 | -0.4460 | No |
| 54 | ARRDC3 |  |  | 2684 | -0.314 | -0.4503 | No |
| 55 | SHPRH |  |  | 2692 | -0.315 | -0.4488 | No |
| 56 | CAMTA1 |  |  | 2697 | -0.315 | -0.4466 | No |
| 57 | ZDHHC17 |  |  | 2718 | -0.320 | -0.4483 | No |
| 58 | CCDC174 |  |  | 2720 | -0.320 | -0.4452 | No |
| 59 | SOX12 |  |  | 2721 | -0.320 | -0.4419 | No |
| 60 | GAS2L3 |  |  | 2755 | -0.327 | -0.4469 | No |
| 61 | MLLT11 |  |  | 2760 | -0.328 | -0.4445 | No |
| 62 | ZNF891 |  |  | 2806 | -0.336 | -0.4524 | No |
| 63 | MFN1 |  |  | 2859 | -0.346 | -0.4620 | No |
| 64 | GNPTAB |  |  | 2862 | -0.346 | -0.4589 | No |
| 65 | MSRA |  |  | 2891 | -0.354 | -0.4623 | No |
| 66 | EAPP |  |  | 2954 | -0.365 | -0.4743 | No |
| 67 | SPPL3 |  |  | 2998 | -0.371 | -0.4813 | No |
| 68 | FARP1 |  |  | 3001 | -0.373 | -0.4779 | No |
| 69 | APBB2 |  |  | 3023 | -0.378 | -0.4793 | No |
| 70 | APPBP2 |  |  | 3031 | -0.380 | -0.4772 | No |
| 71 | FBXL5 |  |  | 3034 | -0.381 | -0.4737 | No |
| 72 | ATXN7L3B |  |  | 3040 | -0.382 | -0.4710 | No |
| 73 | KLF12 |  |  | 3058 | -0.387 | -0.4713 | No |
| 74 | AKAP9 |  |  | 3066 | -0.389 | -0.4691 | No |
| 75 | GOLGA7 |  |  | 3182 | -0.424 | -0.4938 | No |
| 76 | FAM110B |  |  | 3191 | -0.426 | -0.4914 | No |
| 77 | WDR82 |  |  | 3214 | -0.431 | -0.4925 | No |
| 78 | RTN1 |  |  | 3232 | -0.436 | -0.4923 | No |
| 79 | NPAS3 |  |  | 3233 | -0.437 | -0.4877 | No |
| 80 | PDZRN3 |  |  | 3276 | -0.449 | -0.4937 | No |
| 81 | PDZRN4 |  |  | 3279 | -0.450 | -0.4896 | No |
| 82 | SMARCA1 |  |  | 3293 | -0.455 | -0.4881 | No |
| 83 | ANKRD12 |  |  | 3311 | -0.460 | -0.4877 | No |
| 84 | KIFAP3 |  |  | 3312 | -0.461 | -0.4829 | No |
| 85 | NMNAT2 |  |  | 3361 | -0.483 | -0.4900 | No |
| 86 | RPAIN |  |  | 3404 | -0.499 | -0.4955 | Yes |
| 87 | ST8SIA4 |  |  | 3406 | -0.500 | -0.4906 | Yes |
| 88 | SYF2 |  |  | 3419 | -0.509 | -0.4883 | Yes |
| 89 | APC |  |  | 3422 | -0.510 | -0.4836 | Yes |
| 90 | JAKMIP2 |  |  | 3433 | -0.513 | -0.4808 | Yes |
| 91 | ARX |  |  | 3443 | -0.516 | -0.4777 | Yes |
| 92 | PHF21A |  |  | 3449 | -0.520 | -0.4736 | Yes |
| 93 | ARL4D |  |  | 3453 | -0.521 | -0.4689 | Yes |
| 94 | SEMA6A |  |  | 3455 | -0.521 | -0.4638 | Yes |
| 95 | QKI |  |  | 3467 | -0.526 | -0.4611 | Yes |
| 96 | SEL1L |  |  | 3489 | -0.537 | -0.4608 | Yes |
| 97 | GNG2 |  |  | 3543 | -0.567 | -0.4684 | Yes |
| 98 | TCEAL4 |  |  | 3552 | -0.572 | -0.4645 | Yes |
| 99 | CRMP1 |  |  | 3583 | -0.594 | -0.4659 | Yes |
| 100 | NCBP2 |  |  | 3588 | -0.598 | -0.4607 | Yes |
| 101 | AGO1 |  |  | 3611 | -0.609 | -0.4600 | Yes |
| 102 | EPHA3 |  |  | 3616 | -0.614 | -0.4546 | Yes |
| 103 | BLOC1S1 |  |  | 3630 | -0.627 | -0.4514 | Yes |
| 104 | VPS28 |  |  | 3643 | -0.639 | -0.4478 | Yes |
| 105 | BCL11B |  |  | 3656 | -0.651 | -0.4441 | Yes |
| 106 | PAM |  |  | 3685 | -0.671 | -0.4442 | Yes |
| 107 | MAGI2 |  |  | 3695 | -0.681 | -0.4394 | Yes |
| 108 | MPPED2 |  |  | 3701 | -0.685 | -0.4336 | Yes |
| 109 | GAD1 |  |  | 3704 | -0.690 | -0.4269 | Yes |
| 110 | IRGQ |  |  | 3721 | -0.700 | -0.4237 | Yes |
| 111 | ZNF91 |  |  | 3729 | -0.708 | -0.4182 | Yes |
| 112 | CXCR4 |  |  | 3733 | -0.710 | -0.4116 | Yes |
| 113 | DLX6 |  |  | 3735 | -0.711 | -0.4044 | Yes |
| 114 | NRXN3 |  |  | 3738 | -0.714 | -0.3975 | Yes |
| 115 | ZNF704 |  |  | 3750 | -0.729 | -0.3927 | Yes |
| 116 | BCL11A |  |  | 3756 | -0.735 | -0.3864 | Yes |
| 117 | GABARAPL2 |  |  | 3768 | -0.759 | -0.3813 | Yes |
| 118 | SATB1 |  |  | 3776 | -0.767 | -0.3751 | Yes |
| 119 | TNRC6B |  |  | 3781 | -0.771 | -0.3681 | Yes |
| 120 | SOX4 |  |  | 3797 | -0.786 | -0.3638 | Yes |
| 121 | MAPK10 |  |  | 3798 | -0.787 | -0.3556 | Yes |
| 122 | NREP |  |  | 3805 | -0.794 | -0.3489 | Yes |
| 123 | ZNF608 |  |  | 3810 | -0.798 | -0.3416 | Yes |
| 124 | NLGN4X |  |  | 3823 | -0.810 | -0.3362 | Yes |
| 125 | PFN2 |  |  | 3841 | -0.832 | -0.3319 | Yes |
| 126 | CCDC88A |  |  | 3854 | -0.848 | -0.3262 | Yes |
| 127 | SLAIN1 |  |  | 3862 | -0.855 | -0.3191 | Yes |
| 128 | ZEB2 |  |  | 3876 | -0.894 | -0.3131 | Yes |
| 129 | CADPS |  |  | 3877 | -0.894 | -0.3038 | Yes |
| 130 | RBP1 |  |  | 3878 | -0.898 | -0.2945 | Yes |
| 131 | MAF |  |  | 3893 | -0.930 | -0.2884 | Yes |
| 132 | SP9 |  |  | 3898 | -0.942 | -0.2796 | Yes |
| 133 | PBX1 |  |  | 3914 | -0.977 | -0.2733 | Yes |
| 134 | ROBO1 |  |  | 3920 | -1.005 | -0.2641 | Yes |
| 135 | TCF12 |  |  | 3931 | -1.022 | -0.2560 | Yes |
| 136 | DCLK2 |  |  | 3945 | -1.043 | -0.2485 | Yes |
| 137 | CUX1 |  |  | 3955 | -1.079 | -0.2396 | Yes |
| 138 | SOX6 |  |  | 3956 | -1.081 | -0.2284 | Yes |
| 139 | NCAM1 |  |  | 3966 | -1.106 | -0.2192 | Yes |
| 140 | IRF2BPL |  |  | 3968 | -1.110 | -0.2079 | Yes |
| 141 | SOX11 |  |  | 3977 | -1.147 | -0.1980 | Yes |
| 142 | TCF4 |  |  | 3984 | -1.173 | -0.1874 | Yes |
| 143 | DPYSL3 |  |  | 4012 | -1.349 | -0.1802 | Yes |
| 144 | SLCO5A1 |  |  | 4015 | -1.362 | -0.1666 | Yes |
| 145 | CHL1 |  |  | 4019 | -1.379 | -0.1531 | Yes |
| 146 | PRDX2 |  |  | 4021 | -1.396 | -0.1388 | Yes |
| 147 | DLX1 |  |  | 4041 | -1.558 | -0.1275 | Yes |
| 148 | DLX2 |  |  | 4055 | -1.704 | -0.1131 | Yes |
| 149 | TUBB2B |  |  | 4063 | -1.810 | -0.0961 | Yes |
| 150 | NRCAM |  |  | 4076 | -2.179 | -0.0765 | Yes |
| 151 | SNTG1 |  |  | 4086 | -2.546 | -0.0524 | Yes |
| 152 | DLX5 |  |  | 4090 | -2.619 | -0.0259 | Yes |
| 153 | DCX |  |  | 4091 | -2.671 | 0.0018 | Yes |
Table: GSEA details [plain text format]

  

Fig 2: RICHARDS\_NOWAKOWSKI\_SCIENCE\_2017\_IN\_CTX\_MGE1\_UPREG: Random ES distribution      
 Gene set null distribution of ES for **RICHARDS\_NOWAKOWSKI\_SCIENCE\_2017\_IN\_CTX\_MGE1\_UPREG**
